# Do Curriculum-Based Social and Emotional Learning Programs in Early Childhood Education and Care Strengthen Teacher Outcomes? A Systematic Literature Review

**DOI:** 10.3390/ijerph17031049

**Published:** 2020-02-07

**Authors:** Claire Blewitt, Amanda O’Connor, Heather Morris, Aya Mousa, Heidi Bergmeier, Andrea Nolan, Kylie Jackson, Helen Barrett, Helen Skouteris

**Affiliations:** 1 Monash Centre for Health Research and Implementation, Monash University, Melbourne, VIC 3168, Australia; claire.blewitt@monash.edu (C.B.); mandy.oconnor@monash.edu (A.O.); heather.morris@monash.edu (H.M.); aya.mousa@monash.edu (A.M.); heidi.bergmeier@monash.edu (H.B.); 2 School of Education, Faculty of Arts and Education, Deakin University, Geelong, VIC 3220, Australia; a.nolan@deakin.edu.au; 3 Bestchance Child Family Care, Melbourne, VIC 3150, Australia; kjackson@bestchance.org.au (K.J.); hbarrett@bestchance.org.au (H.B.); 4 Warwick Business School, Warwick University, Coventry CV4 7AL, UK

**Keywords:** preschool, kindergarten, social and emotional learning, social and emotional development, teaching quality, teacher-child interaction, pedagogy

## Abstract

There is growing awareness of the benefits of curriculum-based social and emotional learning (SEL) programs in Early Childhood Education and Care settings for children’s social, emotional, and cognitive development. While many SEL programs aim to strengthen teachers’ capacity and capability to foster children’s social and emotional skills, research effort has focused on understanding the impact on child outcomes, with less emphasis on improvement in teaching quality. This systematic literature review examined the effectiveness of universal curriculum-based SEL programs on teacher outcomes. Fifteen studies met inclusion criteria, capturing ten distinct SEL interventions. The findings suggest SEL programs may strengthen teaching quality, particularly the provision of responsive and nurturing teacher-child interactions and effective classroom management. Data were insufficient to ascertain whether participation improved teachers’ knowledge, self-efficacy, or social-emotional wellbeing. The potential pathways between SEL intervention, teaching quality and children’s developmental outcomes are discussed.

## 1. Introduction

Engagement in Early Childhood Education and Care (ECEC) services can strengthen children’s social-emotional and cognitive development, with benefits that persist over time [1]. The quality of these Early Learning programs is an important predictor of language and literacy skill, social-emotional competence, and behavioural engagement [2,3,4,5], particularly for children experiencing economic disadvantage [6,7,8]. 

### 1.1. Importance of High-Quality ECEC for Children’s Social-Emotional Development

The quality of ECEC programs is influenced by the social, emotional, and instructional aspects of children’s interactions with educators and peers (known as process quality) [9]. Process quality is influenced by the physical classroom environment, teacher-child ratio, group size, staff training and qualifications (known as structural quality), as well as teachers’ own personal attributes. For example, high levels of self-efficacy have been associated with positive expectations for children [10], empathy [11], increased use of high-quality practices in preschool rooms [12], and time spent teaching social, emotional, and cognitive skills [13]. Educators’ own social and emotional wellbeing can influence their ability to build strong relationships and facilitate positive outcomes for children [14], and teacher stress has predicted lower levels and less consistent emotional support [15] and lower quality teaching practices [13]. Empirical research and theory emphasize that high-quality teacher-child interactions are especially vital to children acquiring the social-emotional skills necessary to form prosocial relationships and engage in learning [3,4,5,16]. However, studies indicate many children are not consistently exposed to the quality of interactions required for optimal development [17,18,19,20,21,22]. 

### 1.2. Social and Emotional Learning Programs in Early Learning Settings

A growing number of social and emotional learning (SEL) programs designed for Early Learning settings focus on both high-quality teacher-child interactions and targeted pedagogy to nurture children’s social and emotional development. The Collaborative for Academic, Social and Emotional Learning (CASEL) define SEL as the process through which children recognise, understand and regulate their emotions, empathize with the feelings and experiences of others, build and maintain prosocial relationships, establish and achieve positive goals, and make responsible decisions [23]. CASEL propose five competencies, comprising knowledge, skills, and attitude that underpin SEL: self-awareness, social awareness, self-management, relationship skills, and responsible decision-making. (Table 1). The application of SEL approaches in ECEC settings, however, requires careful consideration of the unique developmental characteristics of preschool-aged children, such as the emerging cognitive abilities that underpin social and self-awareness, and the limited ability to regulate behaviour compared to older children [24]. 

Early childhood educators can foster children’s SEL through a variety of approaches including explicit social-emotional skill instruction, child-centered practices and positive classroom management strategies that promote cooperation and prosocial behaviour, and integration within a wider pedagogy [23,25]. Programs that emphasize improving the quality of educator-child interactions, modifying the room environment or processes, or introducing different ways to structure peer interactions have an implicit focus on SEL. In contrast, explicit programs typically include a curriculum-based component, targeting social and emotional skills through instructional practices, modelling, and opportunities for practice across different contexts [23]. SEL programs may be delivered to all children within a group (universal programming) or be offered as an early intervention or treatment approach for children at risk of or experiencing social, emotional, or behavioural difficulties [23]. 

### 1.3. Recent Research Reviews

Most reviews examining the effectiveness of ECEC-based SEL intervention have focused on child outcomes, suggesting a small-to-moderate impact on children’s social-emotional functioning [26,27,28]. A recent systematic review and meta-analysis by our research group of 79 controlled intervention studies (capturing 51 distinct SEL programs) found that children who participated in universal, curriculum-based SEL programs showed significant improvement in social competence, emotional competence, behavioural self-regulation, and early learning skills, and reduced behavioural and emotional challenges post-intervention compared to control group peers [29]. However, researchers have noted the currently limited understanding of specific program components related to positive outcomes [26,29,30].

Recognising that child behaviour is highly influenced by teacher behaviour, many SEL programs aim to influence child outcomes by strengthening teachers’ capacity and capability to implement evidence-based SEL practices with fidelity [31]. Yet, much research effort has focused on understanding the impact of these programs on children, with fewer evaluations addressing teacher-level outcomes. A recent meta-analysis evaluated the impact of teacher training (with and without a curriculum-based component) on both child *and* teacher outcomes, finding training was moderately effective at improving childcare quality, caregiver interaction skill, and children’s social-emotional development [32]. The inclusion of explicit curricula alongside teacher training did not appear to be a significant moderator of program success. However, only five of the 19 studies in this review included a curriculum-based component. To our knowledge, there lacks a synthesis of research on the effectiveness of curriculum-based SEL interventions for teacher-level outcomes. 

### 1.4. Study Aim

Closer examination of the impact of universal curriculum-based interventions (i.e., programs that include explicit SEL skill instruction and are delivered at the class-wide level) on a broad range of teacher-level outcomes is needed to understand the domains in which teachers benefit from SEL programs, and the pathways by which SEL programs can influence children’s developmental trajectories [33]. The aim of the present paper, therefore, was to systematically examine the following research questions: (i) what type of teacher outcomes have been evaluated in studies examining universal curriculum-based SEL programs in ECEC settings, and what measures have been used to capture domains of teacher behaviour, practice, and wellbeing?; (ii) what does the literature reveal about the effectiveness of universal curriculum-based SEL programs in ECEC settings for teacher outcomes?; (iii) are certain program characteristics associated with program benefits?; and (iv) what are the methodological limitations of research examining the impact of universal curriculum-based SEL programs on teacher outcomes, and what recommendations can be made for future research?

## 2. Methods

### 2.1. Search Strategy and Study Selection 

This systematic review was conducted in accordance with the Preferred Reporting Items for Systematic Reviews and Meta-Analyses (PRISMA) guidelines [34]. Three electronic databases, MEDLINE Complete, PsychINFO and ERIC were searched using combinations of the following key terms: intervention*, program*, curricul* and “early learning centre”, “early learning center”, preschool, preschool*, “pre school”, “pre-school”, childcare, “childcare”, kinder*, “pre kindergarten”, “pre-kindergarten”, “pre-K”, “pre K”, “day care”, daycare, “Head Start”, HeadStart and social, emotion*, social-emotional, “SEL”, “self-esteem”, empathy, “emotional intelligence”, “conflict resolution”, “problem solving”, resilien*, aggress*, anxi*, prevent*, externali*, internali*, withdraw* and educator*, teacher*, leader*. Additional articles were identified by scanning reference lists of included studies and relevant systematic reviews. The search aimed to identify peer-reviewed studies that evaluated the impact of universal, curriculum-based SEL programs in ECEC settings on teacher outcomes, published in English between 1999 and 2019. All database searches were carried out between July to August 2019. 

### 2.2. Inclusion and Exclusion Criteria

Studies were assessed against the inclusion and exclusion criteria presented in Table 2. 

### 2.3. Review Procedures and Data Abstraction

**Figure 1:**
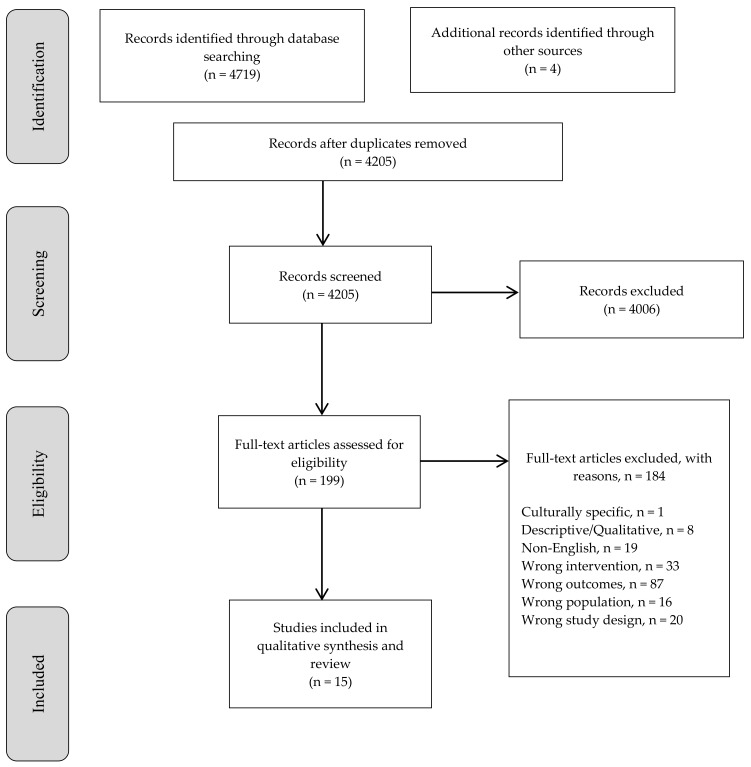
Flow diagram of studies included in review.

The systematic search identified 4205 articles after the removal of duplicates (Figure 1). All titles and abstracts were screened by one author (C.B.), with a second author (A.O.) independently co-screening 10% of the titles and abstracts; agreement for articles to be read in full was 100% after discussion. One hundred and ninety-nine papers were read in full, with 15 included in the review. Two articles that provided data relating to the same study were combined [35,36]. The following pre-specified data were extracted from each study: (i) ECEC setting; (ii) study design; (iii) sample size (number of teachers); (iv) teacher characteristics; (v) type of control group; (vi) SEL program; (vii) program components; (viii) teacher education component where relevant; (ix) outcome, outcome measure and informant (teacher, observer); and (x) findings, including effect sizes where reported by the author.

### 2.4. Quality of Evidence

Study quality was assessed against the Effective Public Health Practice Project (EPHPP) Quality Assessment Tool for quantitative studies with respect to selection bias, study design, confounders, blinding, data collection methods, withdrawals, dropouts, intervention integrity, and analyses [37]. This tool is suitable for randomised, non-randomised, and pre-post designs, and was utilised in our recent review examining the impact of curriculum-based SEL interventions on child outcomes [29]. Components were rated as strong, moderate or weak across each study, based on guidelines in the EPHPP Dictionary and an overall global quality rating was assigned. Studies were rated as strong when no weak ratings were recorded. Those with one weak rating were considered of moderate quality, and two or more weak ratings resulted in an overall weak rating.

## 3. Results

### 3.1. Summary of Included Studies

The pooled characteristics of the 15 studies included in this review are provided in Table 3 and detailed further in Table S1 in the Supplementary File. Table 4 summarizes the teacher-level outcomes, measures, and key findings from each paper. Ten studies were described as randomised or cluster-randomised controlled trials [36,38,39,40,41,42,43,44,45,46,47] and four quasi-experimental trials [48,49,50,51]. Settings included kindergarten [40,41,47], childcare [43,46], preschool [38,39,44,45,48,49,50], Head Start preschool or kindergarten (early childhood education services provided to low-income children and families in the United States) [35,42,44,45,47,51] and early school grades [40,44,47]. In most studies, control group children participated in a business-as-usual ECEC curriculum [n = 13,35,38,39,41,43,44–51], with a smaller group of studies employing active controls including a literacy program [40], and a trust–based relational intervention with a relationship building course and daily activities [42]. Thirteen studies were conducted in the United States, and two in Turkey [38,50], and all were published in the last 15 years. A total of 736 teachers were captured by the studies in this review. One review reported their study was conducted in the kindergarten rooms of four low-income schools, including 327 children, however, did not specify the number of teacher participants [41]. Seven studies (46.7%) were assessed as strong quality [35,39,40,43,44,46,47], two (13.3%) as moderate quality [41,45], and six (40.0%) as weak quality [38,42,48,49,50,51] (Table 4).

### 3.2. SEL Program Characteristics

Ten SEL programs were evaluated within the included studies; five papers examined Preschool Promoting Alternative THinking Strategies (Preschool PATHS) [35,38,41,44,50], two the Second Step Preschool/Kindergarten Social/Emotional Learning Curriculum and Second Step Early Learning Curriculum [45,46] and one each the Incredible Years Dina Dinosaur Social Skills and Problem Solving Curriculum (Incredible Years Curriculum) [47], Tools of the Mind [39], INSIGHTS [40], Strong Start Pre-K [48], OpenMind [42], Responsive Early Childhood Curriculum, plus explicit SEL activities (RECC+) [43], I Can Problem Solve [51] and the Peace Education Foundation Curriculum [49]. INSIGHTS [40] was delivered by a trained facilitator and the Incredible Years Curriculum [47] by the lead researcher, both in partnership with the classroom teacher. All other programs were led by the classroom teacher. With the exception of Tools of the Mind [39] and OpenMind [42], which embedded SEL activities into the curriculum, studies included explicit SEL lessons or activities. As shown in Table 3, most programs provided early childhood educators with training and support during implementation. 

### 3.3. Teacher-Level Outcomes and Measures

The studies captured in this review examined the following teacher-level outcomes: (i) teaching practice and behaviour, including teacher-child interactions; (ii) teacher-child relationship quality; (iii) teachers’ social-emotional wellbeing; and (iv) teacher knowledge of SEL. Eleven studies examined the impact of SEL programs on teaching practice and behaviour, using observational assessments. Measures included the Teacher Style Rating Scale (TSRS) [35,38,50], an assessment of positive discipline, classroom structure and management, emotional communication and support, social awareness and social problem solving; the Classroom Assessment Scoring System (CLASS) [35,39,40] to gauge emotional, organisational and instructional interactions within the classroom; the Teacher Behaviour Rating Scale [43,44], with subscales measuring the quantity and quality of specific teaching behaviours; the Caregiver Interactions Scale [46] addressing the quality of teacher-child interactions across positive, punitive, permissive and detached domains; and the Multiple Option Observation System for Experimental Studies to code teacher-focused behaviours including positive reinforcement/praise, critical statements, and the amount of interaction/involvement with children [47]. The Teacher Coder Impressions Inventory was included in one study to evaluate teaching style across five scales: harsh/critical, inconsistent/permissive, warm/affectionate, social-emotional teaching and effective discipline [47]. 

An overall assessment of classroom quality (including space, personal care routines, language and reasoning, interactions, program structure and parent involvement) using the Early Childhood Environmental Rating Scale-Revised was included in two studies [39,46], and classroom environment quality using the Classroom Atmosphere Rating Scale in another two [38,50]. This measure includes assessment of both teacher responsiveness and supports, and a global measure of child behaviour. Four studies examined specific teaching practices, including the use of scaffolding with the Preschool Classroom Observation Scale [39], literacy instruction with the Supports for Early Literacy Assessment [39], child-directed talk with the Classroom Language and Literacy Environmental Observation [35], and frequency of social-emotional and executive functioning activities using the Social-Emotional and Executive Functioning Classroom Observation Tool [45].

Four studies included a measure of teacher-child relationship quality based on teacher report. Three used the Student-Teacher Relationship Scale [41,48,50], and one the Adult-Child Relationship Scale [43]. Only one author measured teacher’s own social-emotional wellbeing, examining stress levels using the Perceived Stress Scale, and tendency to be mindful with the Five Facet Mindfulness Questionnaire [42]. Finally, two authors assessed teacher knowledge of SEL techniques, including open-ended test questions to assess teachers’ understanding of topics, activities and skill before and after taking part in the Creating Caring Children and Peacemaking Skills for Little Kids/Helping Not Hurting training sessions [49] and teacher dialogue before and after a 13-session college course to support implementation of I Can Problem Solve in Head Start classrooms [51].

### 3.4. Effects of SEL Intervention on Teaching Practice and Behaviours

The four studies that examined the impact of the Preschool PATHS curriculum on teaching practice and behaviour reported improvement post intervention [35,38,44,50]. Intervention group teachers outperformed controls who did not participate in a SEL intervention on a measure capturing effective discipline, emotional communication and support, social awareness and problem solving, and behaviour management in Arda and Ocak [38], and a trend towards improvement on the same measure was observed in another study of 29 Turkish teachers [50], who also found enhanced classroom environment quality in favour of intervention group participants. 

The emotional climate (assessed as emotion expression, emotion regulation and emotional modelling) and effective classroom management subscales of the TSRS suggested greater improvement in teachers who delivered Preschool PATHS at post-intervention compared to a comparison group in Domitrovich and colleague’s study [35]. An intervention effect did not emerge for positive discipline, however intervention teachers scored significantly higher on the proactive/preventive classroom management subscale. PATHS teachers also demonstrated greater emotional support on the CLASS measure. However, this did not reach statistical significance. Analyses of individual subscales however suggested a significant and moderate intervention effect on positive climate, and a borderline significant effect on teacher sensitivity. Improvement in the instructional support scale also reached borderline significance. Groups in this study did not differ on measures of productivity, quality of feedback, concept development or instructional learning formats. Teachers did, however, make more statements and ask more questions than control group peers based on the Classroom Language and Literacy Environmental Observation measure. 

Bierman et al. [36] conducted follow-up assessments at one year post-intervention for 82% of the teachers who implemented the Preschool PATHS curriculum in Domitrovich et al.’s [35] study. Teachers who had delivered Preschool PATHS rated higher on the emotional climate scale and all subscales (emotional expression, emotion regulation, and emotional modelling) of the TSRS and the emotional support scale of the CLASS. Intervention effects favouring PATHS teachers were also reported for the positive discipline scale of the TSRS. The classroom management scale reached borderline significance, however there were no meaningful group differences for instructional support assessed by the CLASS measure. Teachers who participated in PATHS also asked children more general questions one year post-intervention, with differences in the number of statements, contextualized talk, ratings of sensitivity and richness of talk appearing marginally significant in favour of the intervention group.

In a cluster-randomised controlled trial, Lonigan et al. [44] compared a literacy and math-focused preschool curriculum including Preschool PATHS lessons (explicit SEL) and a version where teachers were provided with professional development and guidance on behaviour management but these skills were not the focus of any specific classroom activity (implicit SEL), to a business-as-usual condition. Observations showed that both intervention groups (with and without explicit SEL curricula) made significant improvements in classroom community, use of lesson plans, and team teaching compared to controls, albeit the two intervention groups did not differ significantly from each other on these outcomes. The curricula without explicit SEL lessons appeared to improve teachers’ use of effective discipline strategies, however this did not emerge for the explicit SEL group. The two SEL groups did not differ from controls on measures of teacher sensitivity or learning centres (the provision of engaging and age-appropriate materials linked to learning themes).

Using a similar research design, another study compared the Responsive Early Childhood Curriculum with and without explicit social-emotional classroom activities to a control group receiving no intervention. Childcare teachers in both intervention groups (with and without the explicit SEL component) outperformed comparison group peers on a measure of teacher responsiveness and instruction. The inclusion of explicit SEL activities did not appear to strengthen the intervention effect [43].

Barnett et al. [39] found teachers who delivered Tools of the Mind curriculum demonstrated significantly higher productivity (management of instructional time and routines) compared with control group teachers, with assessment of teacher sensitivity (responsiveness and offering a secure base to children) reaching borderline significance. Teachers also used more scaffolding techniques than controls, provided a richer literacy learning environment, and scored higher on an overall assessment of classroom quality using the Early Childhood Environmental Rating Scale - Revised. Results did not indicate differences between groups on positive, negative, or over-controlling classroom climate, behaviour management techniques, concept development, learning engagement, or quality of teacher feedback. Similarly, teachers who delivered INSIGHTS to Kindergarten and Grade 1 classrooms in the United States offered children higher levels of emotional support post-intervention, after controlling for pre-test score and covariates, compared with attention-control group teachers who provided a literacy program. These effects were moderated by classroom level; the impact appeared more pronounced for first grade teachers and least pronounced for kindergarten educators. Levels of classroom organisation did not differ between groups at post-intervention [40]. 

The Incredible Years Curriculum, delivered in conjunction with the Incredible Years Teacher Classroom Management Program, led to positive improvement in teacher behaviour in a randomised controlled trial of 153 teachers and 1768 children [47]. Multi-level modelling suggested that intervention group teachers became less harsh/critical and inconsistent/permissive, appeared more warm/affectionate, and placed greater emphasis on social-emotional teaching. Improvement in effective discipline appeared to depend on setting; kindergarten and Grade 1 teachers showed greater improvement than Head Start teachers [47]. Similarly, intervention teachers used fewer critical statements with children, with the teachers observed to be the most critical at baseline making the greatest improvement. Intervention effects were not observed for measures of teacher involvement or levels of teacher praise.

Upshur, Wenz-Gross, and Reed [46] evaluated the Second Step curriculum across two annual cohorts in community childcare centres. Intervention teachers in the first cohort did not appear to differ from control peers in the quality of their interactions with children. The second cohort however showed greater improvement in teacher-child interaction skill and effective discipline. These effects remained significant in an adjusted model accounting for covariates and nesting of children within classrooms. Non-significant trends with large effects favouring the intervention classrooms were reported for general supervision and staff-child interactions. The Second Step Early Learning Curriculum combined instruction and activities to improve children’s social-emotional competence and executive functioning. Intervention group teachers implemented significantly more executive functioning activities at post-intervention than control peers, however only one social-emotional outcome (calming down) favoured the intervention group. There were no differences between conditions on the frequency of other SEL activities, including identifying feelings, perspective taking, helping children to understand strong emotions, social problem solving or friendship skills [45].

### 3.5. Effects of SEL Intervention on Teacher-Child Relationship Quality 

Four studies included a teacher-rated assessment of teacher-child relationship quality, a construct closely related to teacher-child interactions [18], with mixed effects reported. Participation in Preschool PATHS did not lead to improvement in teacher-child conflict or closeness, but was associated with increased dependency (an overreliance on the teacher as a source of support) in one study [50]. In another that compared teachers who delivered the Strong Start curricula, a group who delivered Strong Start and two booster lessons, and a control group, all three conditions showed improvement in teacher-child closeness at post-intervention. Further, teachers in the intervention group who did not receive booster lessons reported significantly greater levels of dependency in their relationships with children, while the group with boosters and control peers reported a decrease. The intervention was, however, associated with decreased levels of teacher-child conflict, while conflict in the control group increased. This improvement was most pronounced for teachers that delivered the curricula with two booster lessons [48]. In a randomised controlled trial of Preschool PATHS in a kindergarten setting, intervention teachers reported greater improvement in overall relationship quality, conflict and closeness compared to the control group, however closeness did not remain significant in a propensity score-matched sample controlling for baseline differences [41]. Likewise, Landry et al. [43] found teachers of children aged 2 to 3 years who participated in the Responsive Early Childhood Curriculum with and without an explicit SEL component reported greater improvement in closeness and reduced conflict with children compared to controls.

### 3.6. Effects of SEL Intervention on Social-Emotional Wellbeing 

Only one study considered the impact of SEL on teachers’ social-emotional wellbeing. Jackman and colleagues [42] evaluated the OpenMind curriculum, including child, teacher and parent components. Teachers attended a five-day training course focused on meditation, were requested to meditate for 20 minutes per day and facilitate daily practices with children in their classrooms. Authors revealed that intervention teachers were better able to describe their feelings compared with controls, albeit there was no effect on other aspects of dispositional mindfulness: observing, acting with awareness, non-judging and non-reactivity. Results suggested a slight increase in teacher stress in the intervention group, and a slight decrease in the control group; however, this did not reach statistical significance.

### 3.7. Effects of SEL Intervention on Educator Knowledge of Social-Emotional Learning

Two studies reported improvement in teacher knowledge of SEL following training that accompanied a classroom curriculum. Teachers who attended the Creating Caring Children and Peacemaking Skills for Little Kids/Helping Not Hurting training as part of the Peace Education Foundation program exhibited significant improvement in their knowledge of program concepts between pre- and post-assessment [49]. Similarly, teachers who attended a 13-session college course to support implementation of I Can Problem Solve showed significant improvement in conflict resolution practices [51].

## 4. Discussion

The social-emotional skills that emerge during early childhood are vital for later social-emotional competence [52]. An established body of research evidence highlights the benefits of high-quality ECEC for children’s healthy development [1,53,54,55], and there is increasing focus on programmatic approaches to encourage educators to intentionally foster children’s social and emotional skill growth within the Early Learning environment. Evaluations of SEL programs that target both educator behaviour and child outcomes in ECEC settings suggest benefits for children across developmental domains. Less is known however about the effects of these interventions on teaching quality and practice. To our knowledge, this is the first systematic review to consider the effectiveness of universal curriculum-based SEL interventions in ECEC settings for educator-level outcomes. 

### 4.1. Teacher-Level Outcomes Evaluated in SEL Research 

Studies examined the impact of SEL programs on teaching quality, teaching practice and behaviour, teacher-child interactions and relationship quality. Only one study included a pre-post measure of teacher wellbeing [42], and two assessed educator knowledge [49,51]. Teachers’ beliefs, knowledge, experiences, self-efficacy, mental health, and social-emotional competence directly influence their ability to support children’s social-emotional development [10,11,13,14,15,56]. However, the impact of SEL programs on these personal attributes could not be determined. 

### 4.2. Effectiveness of Universal SEL Programs for Teacher-Level Outcomes

Most evaluations captured in this review reported improvement in at least one aspect of teaching practice as a result of the SEL program. Findings, however, varied substantially across studies and outcome measures. The following programs appeared to strengthen teachers’ emotional support, sensitivity, responsivity, or capacity to create a positive classroom climate: Preschool PATHS [35,38], the Incredible Years Curriculum [47], Tools of the Mind [39], INSIGHTS [40], and RECC+ [43]. This is an interesting finding given the importance of emotional support within ECEC settings. High-quality emotional interactions have been associated with children’s social skills, after adjusting for prior skills, child, family, and program characteristics [57], and higher levels of social competence [58]. Researchers also suggest that emotional support may benefit behavioural engagement, which in turn encourages pre-academic skills [2,59]. 

Responsive caregiving, an important aspect of emotional support, was captured in several studies. Responsivity encompasses educators’ ability to read and respond to children’s cues, and individualize their teaching style to child need [60]. Developmental theory posits that responsivity can encourage attachment between a caregiver and child that fosters positive emotional, social, and cognitive development [61]. However, researchers suggest there can be a tendency for infrequent responsive and cognitively challenging conversations between teachers and children in early childhood settings, especially for children experiencing disadvantage, with some studies reporting that preschool programmes serving low-income communities appear to offer limited opportunities for responsive teacher-child interactions [19,20]. The improvement in observed emotional support from teachers who participated in SEL programs is therefore a promising finding.

Several authors also reported improvement in teachers’ use of positive classroom management and discipline strategies at post intervention [35,36,38,44,46,47]. Behaviour guidance within the early years classroom can strengthen children’s self-regulation. For example, effective classroom management in kindergarten settings has been associated with children’s behavioural and cognitive self-control, behavioural engagement, and reduced duration of off-task behaviour [62]. These findings highlight a potential pathway between SEL intervention and improved child outcomes via teaching practices that promote children’s cooperation and prosocial behaviour. 

While positive intervention effects were reported across several studies, it is important to recognise the time and effort required to participate in professional learning and implement SEL with fidelity may also have a detrimental effect for some educators. For example, Jackman and colleagues found educators who participated in the OpenMind curriculum reported increased stress levels at post-intervention compared to controls, though this did not reach statistical significance [42]. Lonigan et al. [44] compared a lesson-based SEL curriculum (Preschool PATHS) with an implicit model, reporting improvement in teaching practice for educators in both conditions; however, no difference between the implicit and explicit versions. Similarly, Landry et al. [43] found explicit SEL activities did not appear to offer additional benefit beyond a responsive early childhood curriculum. Continued investigation of the benefits of explicit instruction in combination with implicit approaches, for both children and teachers, is needed to ensure educator time and effort is warranted. 

### 4.3. Program Characteristics Associated with Improvement in Teacher-Level Outcomes

SEL programs shared a common goal to strengthen educators’ capability to foster children’s social-emotional skills through explicit and active instruction, modelling, reinforcement, and practice, albeit differing in several respects including the SEL competencies targeted, program intensity and duration, and the extent to which educators’ own social-emotional wellbeing was addressed. Based on the research currently available, it is difficult to specify SEL program characteristics associated with program success. 

A common feature across studies, however, was the professional learning support offered to educators. Many SEL programs paired comprehensive teacher education with regular consultation focused on educators’ knowledge of strengthening social-emotional development in the preschool setting. Research suggests behaviour change in early years settings is most likely when specific training is combined with on-the-job coaching, feedback on observed performance, assistance with planning and implementation, and support with challenges and decision-making [63]. It is possible the sustained support offered as part of SEL interventions prompted teachers’ continued awareness of their teaching practices and interactions, and strengthened their ability to effectively guide children’s attention and behaviour. Specifically, the specialized training prior to SEL intervention may have strengthened teachers’ attitudes, knowledge, and skills by allowing for rehearsal (e.g. through practice, role play) and individualized feedback [64], while the coaching and ongoing support may have increased the likelihood that these skills were embedded into educators’ everyday practice.

### 4.4. Methodological Limitations in the Evidence and Future Recommendations

There are several limitations to the current evidence base that should be acknowledged in interpreting the findings. While many studies were strengthened by the use of controlled designs, validated scales to measure teacher-level outcomes and moderate to high study quality, they varied in the teacher-level outcomes explored, the type of SEL intervention examined, and the form and extent of professional learning offered. Variability in methodologies and measures is indicative of the multi-faceted nature of educational research, and creates complexity when comparing and integrating results across studies, particularly with regards to identifying components of SEL intervention that offer particular benefit for teacher outcomes. The CASEL framework which guided this review emphasizes the importance of a systemic approach to children’s SEL, with integrated and coordinated supports and policies across classroom, service, family, and community levels [23]. It was not possible to ascertain the broader supports offered to educators within the included studies, which may have influenced intervention effects. 

Furthermore, it is possible that teacher-level outcomes may mediate or moderate teachers’ ability to effectively deliver the SEL curriculum. Continued exploration of the linkages between: (i) curriculum-based SEL programs, (ii) professional development and supports, (iii) teacher-level outcomes, and (iv) child outcomes is needed to understand the active ingredients and core components of successful programs. Additionally, investigation into the relative importance and effectiveness of teacher education, SEL curriculum, and the combination of both on teacher and child outcomes would benefit future SEL program development.

Finally, there lacks evidence of the sustainability of improvements in teacher outcomes over time. Only one study included a follow-up assessment [35,36] and the potential benefits of SEL curriculum for ongoing teaching practice is unknown. It is vital that researchers utilise longitudinal methods to better understand the components of SEL program design that lead to social-emotional skill growth, for both teachers and children.

### 4.5. Strengths and Limitations of the Current Review

This review is strengthened by the clearly focused and pre-specified research questions, a thorough and systematic literature search and screening process, detailed data extraction, and assessment of study quality using a validated quality assessment tool. However, the exclusion of unpublished literature and dissertations, studies that were reported in languages other than English, and studies published prior to 1999 means it is possible relevant studies have been missed, potentially introducing bias into the results. Furthermore, due to the heterogeneity in study designs and outcome measures, global effect sizes were not calculated and the review relied on a non-qualitative analysis. As more research becomes available, statistical synthesis of effect sizes across domains of teaching practice, behaviour, and wellbeing may provide further insight [65]. Finally, while the randomised controlled trials captured in this review offer high levels of internal validity, the ecological validity of findings in everyday practice may be limited.

## 5. Conclusions

The findings of this systematic review suggest that universal curriculum-based SEL programs in ECEC settings may strengthen teaching practice and behaviour, particularly the provision of responsive and nurturing teacher-child interactions and effective management of the classroom environment. However, several gaps in knowledge exist. Data were insufficient to ascertain whether participation in SEL programs improved teacher-child relationship quality or teachers’ knowledge, self-efficacy, or social-emotional wellbeing. Further, there was no rigorous evidence of the sustainability of outcomes over time. Due to the diversity in the type of SEL programs and outcome measures captured, it was difficult to identify common features of SEL programs associated with improved teacher-level outcomes. Continued investigation of differential intervention effects (e.g., do certain programs benefit certain groups of educators), the impact of systemic SEL policies and approaches in addition to classroom curricula, and the association between implementation fidelity and both teacher and child outcomes is needed. This review adds to a growing body of SEL research in ECEC settings by exploring the potential pathways between curriculum-based SEL approaches and domains of teaching practice which are critical for children’s developmental trajectories.

## Figures and Tables

**Table 1:** Collaborative for Academic, Social and Emotional Learning (CASEL) Social and Emotional Learning Competencies.

CASEL Competence Domain	Description [23]
Self-Awareness	Recognising emotions, thoughts, strengths and limitations, self-confidence, self-efficacy, understanding of how thoughts, feelings, and actions are connected.
Social Awareness	Understanding and empathizing with others.
Self-Management	Effectively regulating emotions and behaviours, including impulse control and perseverance.
Relationship Skills	Forming and maintaining prosocial relationships, communication, listening, cooperation, managing conflict.
Responsible Decision-Making	Identifying and effectively solving social and behavioural problems, evaluating consequences of actions.

**Table 2:** Inclusion and Exclusion Criteria.

Category	Included	Excluded
Research Design	Randomised controlled trial, quasi-experimental trial with a comparator group (no limits applied on the type of comparison group), or a single-group pre-post design.	Single-case designs.
Research Setting	Centre-based Early Childhood Education and Care settings, including kindergartens, preschools, and child care services for children from birth to 6 years of age.	Family day care. After school-hours care.
Program Type	Curriculum-based Social and Emotional Learning (SEL) program for preschool-aged children that was delivered to all children within the group (universal intervention).	Classroom teacher/educator did not lead or support delivery of the program. Program targeted children experiencing social, emotional, or behavioural difficulties, or children diagnosed with a mental health condition or developmental delay.
	SEL program addressed at least one of the following competencies: self-awareness, self-management, social awareness, relationship skills, and responsible decision making.	
	Program may include other components such as teacher education, coaching, or consultation in combination with the SEL curriculum.	Program focused on teacher education, coaching, or consultation only, without a curriculum component.
Dependent Variable	At least one educator-level outcome was assessed following the intervention. This may include, but was not limited to, teaching quality, practices, or behaviour, the quality of teacher-child interactions or teacher-child relationships, or educator’s own knowledge, self-efficacy or social-emotional wellbeing.	Did not report a teacher-level outcome post intervention.
Publication Status	Published in English between January 1999 and August 2019 and peer-reviewed.	Unpublished reports and dissertations. Articles published in lanugages other than English. Articles published prior to January 1999.

**Table 3:** Pooled Summary of the 15 Studies Investigating the Effect of Universal Curriculum-Based SEL Programs on Teacher-Level Outcomes.

Characteristics	Sample *n* (% Where Applicable)
Geographic Location	
United States	13 (87%)
Turkey	2 (13%)
Date of Publication	
1999–2005	1 (7%)
2006–2010	4 (27%)
2011–2015	6 (40%)
2015–2019	4 (27%)
Study Type	
RCT/CRT	11
QE	4
Sample Size (Teachers)	
0–10	2 (13%)
11–25	2 (13%)
26–50	3 (20%)
51–100	4 (27%)
101–150	1 (7%)
150+	1 (13%)
Unclear	2 (7%)
Intervention Leader	
Teacher	13 (87%)
Teacher and specialist/researcher	2 (13%)
Program Duration (wk)	
<6	1 (7%)
6–12	4 (27%)
13–24	3 (20%)
25–36	2 (13%)
>36 or embedded into pedagogy	4 (27%)
Unclear	1 (7%)
Professional Learning Support	
Training/Workshops	15
Classroom Visits	4
Meeting/Consultation/Coaching	5
Parent Engagement	
Training Sessions/Workshops	4 (27%)
Update/Bulletin/Newsletter	5 (33%)
Not Described	6 (40%)

**Table 4:** Intervention Effects on Teacher-Level Outcomes.

First Author (Year) Citation	Intervention Sample Size Teacher (Children)	Outcome (s)	Instrument (Informant)	Key Findings at Post-Intervention	Quality Assessment
Arda (2012) [38]	Preschool Promoting Alternative THinking (Preschool PATHS) 7 (95)	Teacher Behaviour and Management Techniques: classroom structure and management, discipline, emotional communication and support, social awareness and problem solving, preventing misbehaviour	The Teacher Style Rating Scale (TSRS) (O)	Intervention teachers outperformed control peers on measures of discipline (*p* < 0.05), emotional communication and support *(p* < 0.001), social awareness and problem solving (*p* < 0.001), and preventing misbehaviour (*p* < 0.001). Groups did not differ on classroom structures and management.	W
		Quality of the Classroom Environment: assessment of child behaviours, teacher responsiveness/supports	Classroom Atmosphere Rating Scale (CARS) (O)	Significant group differences on CARS (*p* < 0.001).	
Barnett (2008) [39]	Tools of the Mind 18 classrooms (210)	Global Classroom Quality: space, personal care routines, language and reasoning, teacher-child interactions, program structure, parent involvement	The Early Childhood Environmental Rating Scale-Revised (ECERS-R) (O)	Intervention Group (IG) teachers scored significantly higher than Control Group (CG) peers on ECERS-R (*p* = 0.003). Significant differences reported for activities (*p* = 0.004) and language reasoning (*p* = 0.010) subscales, while interactions (*p* = 0.081) reached borderline significance.	S
		Literacy Environment and Instruction	Supports for Early Literacy Assessment (SELA) (O)	IG teachers scored higher on the SELA (*p* = 0.001) compared to the CG.	
		Use of Scaffolding Techniques	The Preschool Classroom Implementation (PCI) Scale (O)	IG teachers scored higher on the PCI (*p* = 0.002) compared to the CG.	
		Emotional Climate, Classroom Management, Instruction	Classroom Assessment Scoring System (CLASS) (O)	TOOLS classrooms scored significantly higher than CG on productivity (*p* = 0.042) with a trend towards higher levels of teacher sensitivity (*p* = 0.074). Groups did not differ on positive classroom climate, negative climate, over-control, behaviour management techniques, concept development, learning formats and engagement and quality of teacher feedback.	
Cappella (2015) [40]	INSIGHTS 120, 60 in K (~16.57/class)	Emotional Support and Classroom Organisation	Classroom Assessment Scoring System (CLASS) (O)	INSIGHTS teachers showed higher levels of emotional support post intervention compared to attention-control classrooms (*p* < 0.05, *ES* = 0.30). Treatment effect was moderated by grade, and more pronounced for first grade rooms (*p* < 0.05, *ES* = 0.68). No differences between groups on level of classroom organisation.	S
Domitrovich (2009) [35] Bierman (2014) [36]	Preschool PATHS 84 (246)	Emotional Support and Instructional Support	Classroom Assessment Scoring System (CLASS) (O)	CLASS showed moderate differences favouring the IG for emotional support however this did not reach statistical significance (*p* = 0.11, *d* = 0.39,). Significant effect on positive climate item (*d* = 0.61, *p* = 0.04) and a borderline effect on teacher sensitivity (*d* = 0.58, *p* = 0.07) was reported. No group differences on negative climate, over-control and behaviour management subscales. A non-significant trend favouring IG was reported for instructional support (*p* = 0.08, *d* = 0.45).	S
		Teaching Style: positive discipline, classroom management, positive emotional climate	The Teaching Style Rating Scale (TSRS) (O)	The TSRS showed IG improvement on the positive emotional climate subscale (emotion expression, emotion regulation and emotion modelling, *p* = 0.05), and a significant intervention effect for classroom management (*p* = 0.002). There was no difference between groups on positive discipline, however IG teachers scored higher on individual item of proactive/preventive classroom management (*p* = 0.001).	
		Child-Directed Talk: directives, questions, statements, decontextualised talk, richness and sensitivity of teacher’s child centred talk	The Classroom Language and Literacy Environment Observation (CLEO) (O)	IG teachers showed greater linguistic support, made more statements (*p* = 0.001), asked more questions (*p* < 0.001), decontextualised utterances (*p* = 0.005) and engaged in richer and more sensitive talk with children (*p* = 0.004). Effect sizes ranged from *d* = 0.67 to *d* = 0.89. No difference between groups on directives.	
Fishbein (2016) [41]	Preschool PATHS 4 schools (327)	Student-Teacher Relationship: closeness, conflict	Student-Teacher Relationship Scale (T)	Greater improvement in IG in Total Score (*p* < 0.001), closeness (*p* < 0.001) and conflict (*p* < 0.05) subscales.	M
Gunter (2012) [48]	Strong Start Pre-K 4 (84)	Student-Teacher Relationship: closeness, conflict, dependency	Student-Teacher Relationship Scale (T)	Total score increased in both IGs, however only reached statistical significance for the IG + booster lesson group (*p* < 0.05, *d* = 1.20). Both IG groups showed decreased conflict (*p* < 0.05, d = 0.43 for intervention and 0.67 for intervention + booster), while conflict in the CG increased. CG and intervention + booster groups increased level of closeness, with greatest improvement in the intervention + booster condition (*p* < 0.05, *d* = 1.35). The IG group without boosters showed increased dependency (*p* < 0.05, *d* = 0.43), while IG + boosters and CG showed decline.	W
Jackman (2019) [42]	OpenMind (OM) Curriculum 27 (262)	Tendency to be mindful	Five Facet Mindfulness Questionnaire (FFMQ) (T)	Groups differed on the Describe subscale of the FFMQ. IG scores improved from baseline to post-intervention while scores decreased for CG (*p* < 0.05). There was no difference between groups on other subscales (observe, act with awareness, non- judgmental, non-react).	W
		Perceived stress	Perceived Stress Scale-10 (T)	IG showed slight increase in teacher stress between baseline (*M* = 20.33, *SD* = 1.58) and post-intervention (*M* = 21.0, *SD* = 2.24), while CG showed a slight decrease between baseline (*M* = 21.14, *SD* = 2.12) and post-intervention (*M* = 20.42, *SD* = 2.30).	
Landry (2014) [43]	Responsive Early Childhood Curriculum (RECC) plus explicit social-emotional activities 65 (542)	Teacher Behaviour: teacher responsiveness and instruction	Teacher Behaviour Rating Scale (TBRS) (O)	IG showed significantly greater improvement than controls for the average of all TBRS subscales (*p* < 0.0001, *ES* = 1.04). The following subscales reached statistical significance: classroom community (*p* = 0.009, *ES* = 0.61), oral language (*p*= 0.011, *ES* = 0.79), learning centres (*p* ≤ 0.0001, *ES* = 1.74), book reading (*p* = 0.001, *ES* = 1.35), written expression (*p* = 0.005, *ES* = 1.23), print and letter (*p* = 0.0002, *ES* = 1.35), and lesson plans (*p* < 0.0001, *ES* = 1.65). Groups did not differ on subscales relating to sensitivity, discipline, phonological awareness, mathematics, portfolios and team teaching. Both RECC and RECC+ groups scored higher than controls, and did not differ from each other. At post-intervention, total score and 8/13 subscale scores for RECC and RECC+ groups were between medium-low and medium-high quality. In CG, only 3/13 subscales reached the medium-low quality rating.	S
		Teacher-Child Relationship: closeness, conflict	Adult-Child Relationship Scale (T)	Average closeness for RECC and RECC+ was greater than controls (*p* = 0.0065, *ES* = 0.42). Teacher child conflict in RECC and RECC+ was lower than controls (*p* = 0.011, *ES* = −0.49).	
Lonigan (2015) [44]	Preschool PATHS 110 (855)	Teacher Behaviour and Classroom Characteristics	Teacher Behaviour Rating Scale (TBRS) (O)	Teachers in the Explicit SEL group scored higher than controls on the following classroom characteristics: classroom community (*p* < 0.01, *ES* = 0.73), lesson planning (*p* < 0.001, *ES* = 1.0) and team teaching (*p* < 0.01, *ES* = 0.77). The implicit SEL group outperformed CG on the following subscales: classroom community (*p* < 0.01, *ES* = 0.85), discipline (*p* < 0.05, *ES* = 0.48), lesson planning (*p* < 0.01, *ES* = 0.97) and team teaching reached borderline significance (*p* < 0.01, *ES* = 0.49). Explicit and implicit groups did not differ from each other. No intervention effects were reported for teacher sensitivity or learning centres. On specific instructional activities, Explicit SEL group outperformed CG on book reading (*p* < 0.01, *ES* = 0.87), oral language (*p* < 0.05, *ES* = 0.57) and math activities (*p* < 0.05, *ES* = 0.63). The implicit SEL group outperformed controls on book reading (*p* < 0.001, *ES* = 0.87), oral language (*p* < 0.05, *ES* = 0.55), phonological awareness (*p* < 0.05, *ES* = 0.52), and math activities (*p* < 0.01, *ES* = 0.70). Explicit and implicit SEL groups did not differ from each other. No intervention effects were recorded for print activities or writing activities.	S
Pickens (2009) [49]	The Peace Education Foundation (PEF) Socio-Emotional Development Programme 21 (296)	Assessment of educator knowledge following two training workshops: Creating Caring Children (CCC) and Peacemaking Skills for Little Kids/Heling not Hurting: Teaching the I-Care Rules Through Literature (PSLK-HNH)	CCC: 10 open-ended questions (T)	CCC: Significant improvement from baseline (*M* = 26.5) to post (*M* = 43.5, *p* < 0.001).	W
			PSLK-HNH: 21 open-ended questions (T)	PSLK-HNH: Significant improvement from baseline (*M* = 11.46) to post (*M* = 22.08, *p* < 0.001).	
Seyhan (2017) [50]	Preschool PATHS 29 (565)	Quality of the Classroom Environment: includes assessment of child behaviours and teacher responsiveness/supports for child	Classroom Atmosphere Rating Scale (CARS) (O)	Intervention teachers showed greater improvement on CARS compared to controls *(p* < 0.01).	W
		Teacher Behaviour and Management: classroom structure and management, discipline, emotional communication and support, social awareness and social problem solving, preventing misbehaviour	The Teaching Style Rating Scale (TSRS) (O)	Group difference in favour of IG reached borderline significance (*p* = 0.06).	
		Student-Teacher Relationship: closeness, conflict, dependency	Student-Teacher Relationship Scale (STRS) (T)	No differences between groups on conflict and closeness subscales of the STRS. Teachers in the intervention group reported greater dependency in their relationships with children (*p* < 0.001) compared to the CG.	
Upshur (2017) [45]	Second Step Early Learning Curriculum 31 (492)	Frequency of Teacher-Led Social-Emotional (SE) and Executive Functioning (EF) Activities	Social-Emotional and Executive Functioning Classroom Observation Tool (SEEF) (O) (based on sample of 8 IG and 8 CG classrooms)	Teachers in the IG implemented significantly more EF activities: attention and engagement *(p* < 0.01), thinking ahead and thinking back (*p* < 0.01), think time (*p* < 0.01), encouraging participation (*p* < 0.01), specific reinforcement (*p* < 0.001) and overall attentiveness (*p* < 0.05). Effect sizes >1.0. Only one SE item favoured IG: calming down (*p* < 0.001). No difference was observed between groups on identifying feelings, perspective taking, understanding strong emotions, social problem solving or friendship skills activities.	M
Upshur (2013) [46]	Second Step Preschool/ Kindergarten Social/Emotional Learning curriculum 56 (341)	Interaction: discipline, general supervision, staff-child interactions	Early Childhood Environment Rating Scale Revised (ECERS-R), Interaction Scale (O)	In Year 1, groups did not differ on any measures. However, effect sizes favoured intervention classrooms in the medium to high range for ECERS-R interaction scale (*d* = 0.35), and ECERS-R interaction items: discipline (*d* = 0.83) and general supervision (*d* = 0.32). In Year 2, IG showed greater improvement in ECERS-R interaction scale (*p* < 0.05, *d* = 1.81) and discipline (*p* < 0.01, *d* = 2.44). General supervision (*p* < 0.10, *d* = 1.78) and staff-child interactions (*p* < 0.10, *d* = 1.49) reached borderline significance. Results remained significant after adjustment for covariates.	S
		Quality of Teacher Interaction Skill: positive, punitive, permissive, detached	Caregiver Interaction Scale (CIS) (O)	Change scores did not differ between groups in Year 1. In Year 2, teacher interaction skills remained stable for the IG and decreased significantly in CG (*p* = 0.03, *d* = 1.74).	
Vestal (2004) [51]	I Can Problem Solve 11 (64)	Perceptions and Practices in Relation to Conflict	ICPS dialogue (T)	Teachers used more ICPS dialogue after training and decreased their non-ICPS dialogue (*p* < 0.05). ICPS dialogue also increased from baseline to post-intervention (*p* < 0.05).	W
Webster-Stratton (2008) [47]	The Incredible Years Dina Dinosaur Social Skills and Problem Solving Curriculum 153 (1768)	Teacher Behaviour: positive reinforcement, critical statements, amount of interaction with children	Multiple Option Observation System for Experimental Studies (MOOSES) (O)	Based on MOOSES, a reduction in critical statements favoured IG. The more critical the teacher was initially, the more the score improved at post. No other constructs reported significant effects.	S
		Teaching Style and Classroom Management: harsh/critical, inconsistent/permissive, warm/affectionate, social/emotional teaching, effective discipline	Teacher Coder Impressions Inventory (TCI) (O)	After controlling for covariates, IG teachers were less harsh/critical (*ES* = 0.67), and inconsistent/permissive (*ES* = 0.63), more warm/affectionate (*ES* = 0.51) and placed more emphasis on social-emotional teaching (*ES* = 0.96). Main effects for effective discipline did not emerge, but intervention effect depended on the grade of the teacher: Kindergarten and Grade 1 teachers showed higher levels of effective discipline than Head Start teachers.	
		Quality of the Classroom Atmosphere: includes assessment of child behaviours and teacher’s classroom management	Classroom Atmosphere Measure (O)	Greater improvement in IG’s classroom atmosphere compared to CG (*ES* = 1.03).	

Note: CG = Control Group, IG = Intervention Group, M = Moderate, O = Observer, S = Strong, T = Teacher, W = Weak.

## Supplementary Materials

The following are available online at https://www.mdpi.com/1660-4601/17/3/1049/s1, Table S1: Characteristics of Included Studies.
