# Supplementary material for: Do Curriculum-Based Social and Emotional Learning Programs in Early Childhood Education and Care Strengthen Teacher Outcomes? A Systematic Literature Review
Source: Int J Environ Res Public Health. 2020 Feb 7;17(3):1049. doi: 10.3390/ijerph17031049 (PMC7036843; doi:10.3390/ijerph17031049)
Supplement: Supplementary file 1 [file ijerph-17-01049-s001.pdf]

International Journal of Environmental Research and Public Health

MDPI

Supplementary Materials: Do Curriculum-Based Social and Emotional Learning Programs in Early Childhood Education and Care Strengthen Teacher Outcomes? A Systematic Literature Review

Table S1. Characteristics of Included Studies.

First Author (Year)	Country	Setting	Study Design	n Teacher (Child)	Control	Intervention	Intervention Leader	SEL Program Duration	Training Component	Parent Component
Arda (2012)	Turkey	P	RT	7 (95)	BAU	Preschool PATHS	T	44 lessons/9 weeks	Teacher education before program, weekly training during implementation	Not described
Barnett (2008)	USA	P	RCT	18 classrooms (210)	Curriculum developed by local teachers	Tools of the Mind	T	Embedded	4 days training, 30 min weekly classroom visits, 0.5 day and 5 x 1 hr meetings	Not described
Cappella (2015)	USA	K, G1	SRT	120, 60 in K (~16.57/ class)	Literacy program	INSIGHTS	F, T	Weekly lesson (45 min)/10 weeks	10 x 2-hr sessions	10 x 2-hr sessions
Domitrovich (2009)<br><br>Bierman (2014)	USA	HS	RCT	84 (246)	BAU	Preschool PATHS (as part of REDI)	T	33 weekly lessons plus extension activities	4 days training, weekly in-class support	Not described
Fishbein (2016)	USA	K	RCT	4 schools (327)	BAU	Preschool PATHS	T	2 lessons per week (20 min)/22 weeks	2-day training, weekly consultation (2-3 hr) in each classroom	Frequent updates
Gunter (2012)	USA	P	QE<br>CG, IG, IG+2 booster lessons	4 (84)	BAU	Strong Start Pre-K	T	2 lessons per week/6 weeks	1 hr training and Strong-Start manuals	Bulletin for each lesson

Jackman (2019)	USA	HS -P	RCT	27 (262)	High Scope, Trust-Based Relational Intervention, SEL<br><br>5-day relationship building course, daily activities<br><br>Parent training on relationship building practices	OpenMind (OM) Curriculum	T	Embedded	5-day mindfulness training, meditation for 20 mins/day	3 x 2hr mindfulness-based training sessions
Landry (2014)	USA	CC	RCT<br>CG, RECC, RECC<br>+ explicit social-emotional activities	65 (542)	BAU	Responsive Early Childhood Curriculum (RECC) plus explicit social-emotional activities	T	Daily, 36 weeks	Training (4 x 6-7 hr), weekly coaching support, teacher manuals	Parent newsletters
Lonigan (2015)	USA	P, HS, PS	CRT<br><br>CG, academic skills focused curriculum with explicit SEL, academic skills focused curriculum with implicit SEL	110 (855)	BAU	Preschool PATHS	T	1-2 times per week/year	Teacher manuals, 8-day training, explicit group received 3 half day training sessions focused on SE activities, 9 monthly coaching sessions (3hr)	Not described
Pickens (2009)	USA	P	CT<br>Pre-post measures relating to teacher knowledge	21 (296)	BAU	The Peace Education Foundation (PEF) Socio-Emotional Development Programme	T	Year	2 days training, 4-6 technical assistance visits	3-hr workshop

International Journal of *Environmental Research and Public Health*

MDPI

Seyhan (2017)	Turkey	P	QE	29 (565)	BAU	Preschool PATHS	T	33 lessons (15-20 min)/ 9 weeks	Previous PATHS training, re-training, weekly support meetings	Not described
Upshur (2017)	USA	HS, P	CRT	31(492)<br><br>Classrooms participated for 2 years	Creative Curriculum or Head Start Frameworks	Second Step Early Learning Curriculum	T	Daily activity 5-7 mins, and integrated within other curriculum requirements	Year 1: 7 x monthly 2-hr training. Year 2: 5 group training sessions.<br>Monthly visits including observation, coaching and written feedback, SSEL curriculum kit	Parent handouts
Upshur (2013)	USA	CC	CRT	Year 1: 30, Year 2 26. >50% teachers participated in both years (341)<br><br>Classrooms participated for 2 years	Creative Curriculum	Second Step Preschool/ Kindergarten Social/Emotional Learning curriculum	T	4 lessons per week (15 min)/22 weeks	Some participants attended workshop, 7 monthly (2 hr) training sessions in Year 1, 5 bi-monthly sessions in Year 2	Four to six parent group sessions/yr
Vestal (2004)	USA	HS	QE<br>Pre-post measures relating to teacher knowledge	11 (64)	BAU	I Can Problem Solve	T	2 months	13-session college-level course (40hr)	Not described
Webster-Stratton (2008)	USA	HS, K, G1	RCT	153(1,768)	Head Start and elementary school curriculum	Incredible Years Dina Dinosaur Social Skills and Problem-Solving Curriculum	T, R	2 lessons per week (15–20-min) followed by 20 minutes of small group skill practice/30 lessons in total	IY Teacher Training: 4 days (28 hours) of training spread across monthly workshops, manuals	Letters, weekly homework for parents to complete with children

Note: BAU=Business as usual, C-RCT = Cluster Randomised Controlled Trial, CRT=Classroom Randomised Trial, CC=Child Care, CG=Control Group, CT=Controlled Trial, F=Facilitator, G1= Grade 1, HS=Head Start, IG=Intervention Group, K=Kindergarten, P=Preschool, PS=Public School, QE=Quasi-Experimental, R=Researcher, RCT=Randomized Controlled Trial, RT=Randomized Trial, SRT= School Randomized Trial, T=Teacher.
